# Impact of urban green spaces on mental restoration in older adults: a perspective based on subjective perception

**DOI:** 10.3389/fpubh.2025.1687874

**Published:** 2025-11-17

**Authors:** Jianjian Zhang, Ziyi Tan, Fanyue Bu

**Affiliations:** College of Art and Design, Nanjing Tech University, Nanjing, Jiangsu, China

**Keywords:** mental restoration, older adults, urban green space, perceived sensory dimensions, environmental features

## Abstract

**Introduction:**

This study investigates the impact of urban green spaces (UGSs) on mental restoration among older adults. It contributes to the development of age-friendly urban green spaces that are more inclusive and promote health.

**Materials and methods:**

Data were collected via surveys conducted in five distinct UGSs in Nanjing, China. The Perceived Sensory Dimensions (PSDs) were used to evaluate the qualities of these green spaces, while the Perceived Restorativeness Scale (PRS) assessed older adults' mental restoration. Multivariate linear regression analyses were conducted to examine the effects of UGS quality on mental restoration in older adults.

**Results:**

Four dimensions of the PSDs—culture, social, rich in species, and serene—had a positive effect on mental restoration in older adults. Gender-specific analysis showed that culture, rich in species and serene, positively impacted mental restoration in both genders. Conversely, the social dimension had a positive effect on older men, while refuge positively affected older women. Age-specific analysis showed that culture, social, and serene positively influenced mental restoration among participants aged 60–79 years, while species richness positively influenced mental restoration among those aged 60–69 years.

**Conclusion:**

Our findings reveal that specific environmental features of UGSs positively influence mental restoration in older adults. Moreover, the effects of environmental features on mental restoration varied by age and gender. Based on these findings, we propose four key considerations for designing UGSs.

## 1 Introduction

The rapid increase in the older population is becoming an urgent concern, presenting aging-related challenges. Among these, geriatric mental health issues have emerged as a significant societal concern (1). Current estimates indicate that approximately 14% of adults aged 60 and older worldwide are affected by mental disorders (2). The 2019 Global Health Estimates (GHE) report states that mental health conditions account for 10.6% of total disability among older adults. Furthermore, the 2019 GHE reveals that approximately 27.2% of global suicide deaths occur among individuals aged 60 and older (3). According to the 2019 China Health and Retirement Report, 33.1% of adults aged 60 and above in China are at high risk for depression (4). Due to limitations in pension and healthcare systems, older adults in China are particularly vulnerable to psychological issues when confronting the challenges of aging (5).

Urban green spaces (UGSs) are components of a “man-made environment” that incorporate natural elements and are primarily used to provide space for relaxation and leisure activities. Additionally, UGSs provide essential ecosystem services, including urban parks, gardens, greenways, etc. (6). Among them, urban parks are a common type of UGS and play a significant role due to their diverse functions, strong appeal, and extensive service coverage. Recent studies have shown that UGSs are beneficial for mental health, as they can help alleviate stress and anxiety (7) and enhance mood and self-esteem (8). In China, UGSs serve as primary venues for outdoor activities among older adults (9). Investigating the impact of these spaces on mental restoration in older adults is essential for improving their mental health.

Since the 1980s, two main theories have guided research on restorative environments: Ulrich's Stress Reduction Theory (SRT) and Kaplan's Attention Restoration Theory (ART) (10). SRT posits that emotions are central to the relationship between the environment and human recovery, arguing that restorative environments balance physiological disorders by generating positive emotions that displace negative ones (11, 12). When individuals experience positive emotional alterations, previously declined cognitive functions may be restored (13).

ART adopts a cognitive framework to elucidate the restorative process. This theory distinguishes between directed and involuntary attention (14). Many activities require sustained attention, which can exhaust directed attention. When directed attention is reduced due to overuse, mental fatigue ensues. The theory suggests that directed attention is best restored through exposure to involuntary attention sources. Natural environments attract people's attention involuntarily yet in a low-demanding manner, promoting emotional improvement and allowing the restoration of attention resources, particularly directed attention (15). According to ART, natural environments facilitating the restoration of directed attention must meet four criteria: (1) being away, allowing individuals to disengage from mental activities requiring sustained attention; (2) fascination, containing captivating objects and processes; (3) extent, being rich and coherent enough to constitute an alternate world; and (4) compatibility, aligning with an individual's intended activities and preferences (14). Hartig developed the PRS to measure the restorative potential of environments along four dimensions (16).

Subsequent studies further explored the relationship between UGS characteristics and mental restoration. Nordh et al. found that grass, shrubs, trees, and water were positively correlated with mental restoration, and that seating promoted restorative experiences (17–19). Peschardt and Stigsdotter found that areas in parks with sunlight, shade, vegetation, and terrain variations could foster mental restoration (20). Lorenzo et al. found a strong association between vegetation abundance in UGSs and mental restoration (21). Liu et al. found that shrubs and water positively affected mental restoration (22).

Current studies further explore the features of UGSs related to mental restoration. Zhang et al. found that spatial openness emerged as the most significant positive factor for both restorative quality and mental health (23). In addition, a variety of mixed vegetation clusters, such as groups of shrubs and trees, were found to be highly important for mental restoration (23, 24). Huang and Wu's research indicated that the “natural elements” and “cultural identifiers” in green space had a positive effect on mental health (25). In addition to the features of UGSs, some scholars conducted research on the mechanisms by which UGSs influence mental health and found that social interaction and outdoor physical activity play a potential mediating role in the association between UGSs and symptoms of depression and anxiety (26). Sense of neighborhood safety and neighborhood cohesion also moderated the influence of UGSs on mental health (27).

Some studies specifically addressed mental restoration among older adults, revealing that interaction with urban natural environments could alleviate physical and cognitive decline and enhance mental wellbeing (28, 29). Additional studies identified factors such as sky view, boundary enclosure, green view, the number of seats, and physiological equivalent temperature in UGSs as influential on mental restoration in older adults (30). Furthermore, exposure to walkable areas, water features, and commercial, recreational, and cultural attractions was associated with improved mental health among older adults (31).

However, certain limitations exist within studies examining the impact of UGSs on mental restoration. First, findings have produced conflicting views regarding which characteristics of green space influence mental restoration, leading to confusion in both academic research and design practice. Second, these studies often overlook potential differences in the mental restoration effects of green spaces on older adults across age groups and gender. Moreover, current studies predominantly rely on objective indicators of green space characteristics to establish a link between green space and mental restoration in older adults. While objective measurements can capture actual environmental conditions, they may neglect users' subjective experiences, leading to evaluations that are misaligned with their true perceptions. For instance, Prins et al. noted that perceived accessibility significantly impacted physical activity, compared to the objective number of parks and sports facilities near respondents' residences (32). Similarly, Gebel et al. found that even in areas with high objective walkability scores, residents might perceive the environment as unsuitable for walking (33). Hao et al. also found that people's mental restoration from plant diversity depended more on their perception of it than on the actual species richness (34). Such discrepancies between perception and objective reality may be particularly pronounced among older adults, who are often more sensitive to environmental details than younger individuals (35, 36).

Thus, the present study uses the Perceived Sensory Dimensions (PSDs) to more accurately capture respondents' environmental perception experiences and assess green space characteristics. The Perceived Restorativeness Scale (PRS) serves as a measurement tool for the restorative potential in older adults. This research aims to investigate the impact of UGSs on mental restoration in older adults by addressing the following three questions:

(1) Which perceptual properties of UGSs affect the mental restoration of older adults?
(2) Do the impacts of these perceptual properties among older adults vary by gender?
(3) Do the impacts of these perceptual properties among older adults vary by age?

This study provides important empirical evidence to advance United Nations Sustainable Development Goal 3 (SDG 3), which aims to ensure healthy lives and promote wellbeing for all at all ages. Meanwhile, it helps to accurately optimize the corresponding environmental characteristics in UGSs by exploring the environmental needs for mental restoration among older adults of different genders and ages. This will thereby enhance older adults' mental health more equitably and effectively and provide an operational environmental support pathway for SDG 3.

## 2 Materials and methods

### 2.1 Perceived sensory dimensions

The current study used the PSDs to assess the characteristics of green space environments. Recognizing that individuals form comprehensive experiences and understandings of their environment through the synergistic action of multiple senses (37), Grahn and Stigsdotter proposed using PSDs as factors for assessing qualities related to the recovery of UGSs in 2010 (38). They conducted a study mailing questionnaires to 2,200 randomly selected respondents across nine Swedish cities to investigate preferences for UGS qualities and self-reported health status. From the 953 valid responses received, factor analysis identified eight primary environmental preference factors: nature, culture, social, serene, space, prospect, refuge, and rich in species. These factors engage multiple senses and are strongly linked to an individual's mental restoration, which is why they are termed PSDs. The PSDs are derived through a bottom-up methodology based on individuals' experiences and perceptions of environmental characteristics, rendering them particularly suitable for on-site assessments of UGS qualities (39).

Since 2010, approximately 100 studies employing PSDs have been conducted globally, including in Europe (40–42), North America (43), and Asia (44–46). Findings from these studies substantiate the classification of the eight PSDs and maintain cross-cultural consistency (47). Furthermore, the application of PSDs in research on health-promoting natural environments has been validated, supporting their effectiveness in assessing the restorative qualities of green spaces in diverse settings (48, 49).

To enhance understanding among Chinese older adults, descriptions were added for each dimension based on Grahn and Stigsdotter (38) and Luo et al. (50). Respondents rated the PSDs of UGSs using a 7-point Likert scale from 0 “complete disagreement with the factors” to 6 “complete agreement with the factors” (Table 1).

**Table 1:** Evaluation of perceived sensory dimensions.

**Dimension**	**Description**	**Scale**						
Nature	Here is nature and wilderness.	0	1	2	3	4	5	6
Culture	There are many artificial element decorations here.	0	1	2	3	4	5	6
Prospect	Here is an open space with a wide view.	0	1	2	3	4	5	6
Social	Here is an environment suitable for social activities.	0	1	2	3	4	5	6
Space	This is a spacious and undisturbed environment.	0	1	2	3	4	5	6
Rich in species	Here is an abundance of plants and animals, like birds, insects, etc.	0	1	2	3	4	5	6
Refuge	Here is an enclosed and safe environment.	0	1	2	3	4	5	6
Serene	Here is a silent and peaceful environment.	0	1	2	3	4	5	6

Definitions of each dimension based on Grahn and Stigsdotter (38) and Luo et al. (50).

### 2.2 Perceived restorativeness scale

Based on ART, Hartig proposed the PRS, which has been widely applied in numerous studies assessing environmental restoration quality (51, 52). A series of simplified versions of the PRS scale was developed in subsequent empirical studies to improve the efficiency of the assessment. The most representative is PRS-11, revised by Pasini et al. (53). This scale comprises 11 questions across the dimensions of Fascination, Being Away, Coherence, and Scope. PRS-11 was designed to evaluate the restorativeness of environments while minimizing participant fatigue, thereby enhancing research efficiency. The modified scale has also demonstrated cross-national and cross-gender invariance (54). However, a pre-survey noted that the 11 questions posed a level of complexity for older adults, potentially leading to fatigue and reluctance to complete the questionnaire.

Following this, we referenced studies by Celikors and Wells (55) and Ma et al. (56) and simplified the scale. In their study, Celikors and Wells selected the most descriptive phrases for each of the four restorative qualities of PRS-11 based on the descriptions and functions of each restorative dimension of the scale. They then calculated intraclass correlation coefficients for the four restorative qualities. The results showed that participants had acceptable agreement on the ratings of these restorative qualities. Ma et al. adopted the simplified version of the PRS-11 proposed by Celikors and Wells and demonstrated that this simplified scale had good reliability and validity in practical application in China. Therefore, we adopted this simplified scale in the formal survey, with responses scored on a 5-point scale ranging from “Strongly Disagree” (1 point) to “Strongly Agree” (5 points) (Table 2).

**Table 2:** Evaluation of the perceived restorativeness scale.

**Restorative quality**	**Description**	**Scale**				
Fascination	In this place, my attention is drawn to many interesting things	1	2	3	4	5
Being-away	To stop thinking about the things that I must get done, I like to go to places like this	1	2	3	4	5
Coherence	It is easy to see how things are organized in this place	1	2	3	4	5
Scope	This place is large enough to allow exploration in many directions	1	2	3	4	5

Definition of each restorative quality based on Celikors and Wells (55) and Ma et al. (56).

### 2.3 Study sites

**Figure 1:**
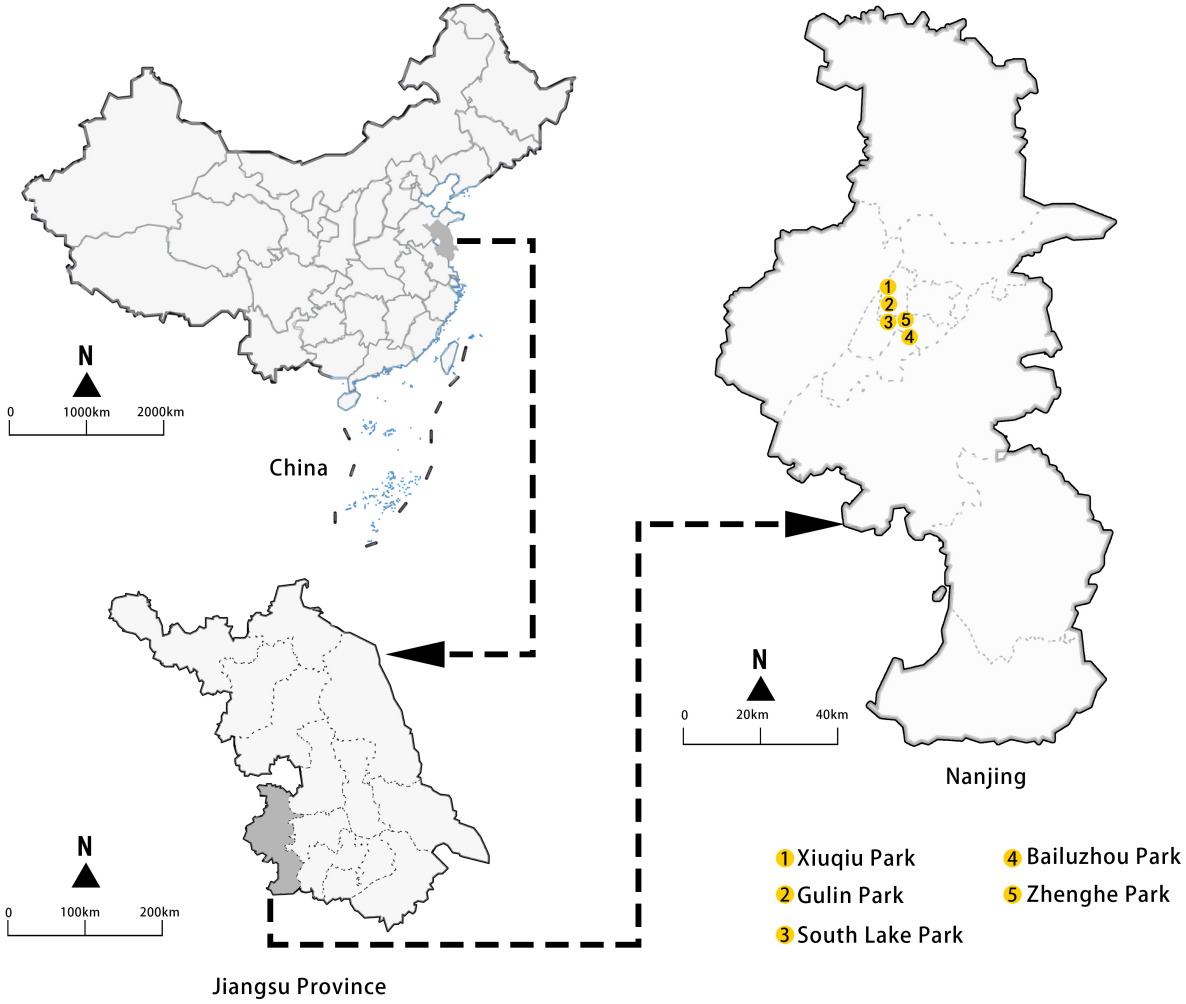
Study sites.

Nanjing, a city in eastern China, is experiencing a growing aging population. According to the Seventh National Population Census Bulletin, individuals aged 60 and above constitute 18.98% of Nanjing's total resident population, indicating a slightly higher level of aging than the national average (57). For this study, five medium-scale public green spaces in Nanjing's inner-city districts were selected for data collection. These included Xiuqiu Park, Gulin Park, South Lake Park, Bailuzhou Park, and Zhenghe Park (Figure 1). The selection was based on the following considerations: (1) these UGSs were adjacent to urban residential areas, providing convenient access for local older adults, thereby facilitating sample collection; and (2) there were diverse landscape features (such as lawns, trees and shrubs, lakes, squares, artificial decorations, and so on) that met the basic requirements of PSDs in these UGSs (Table 3).

**Table 3:** Size and photos of study sites.

**UGS**	**Size of the park**	**On-site photos**	
Xiuqiu Park	10 hm^2^	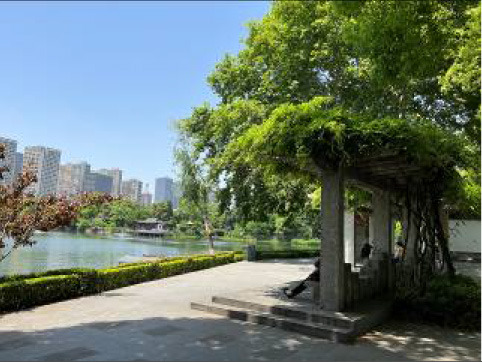	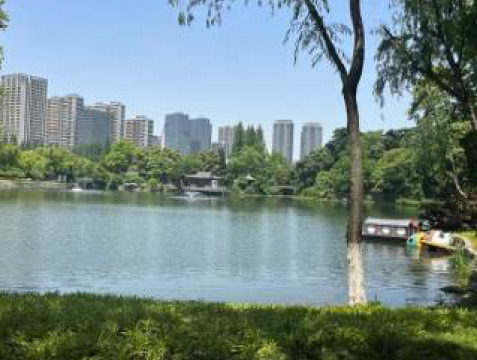
Gulin Park	27 hm^2^	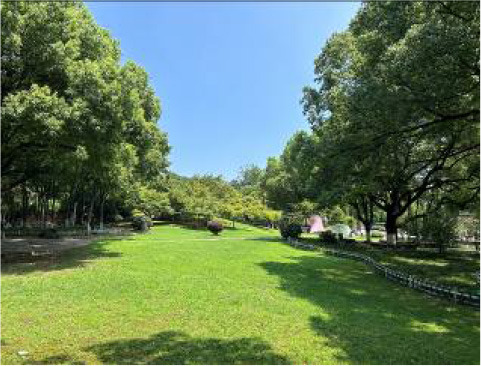	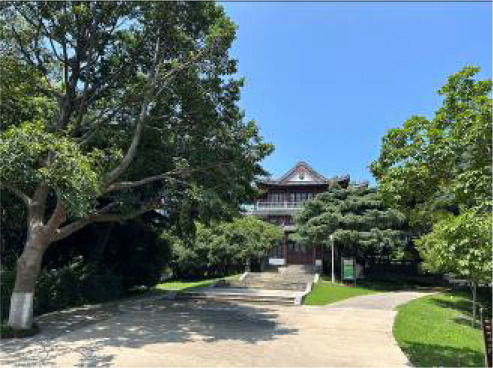
South Lake Park	15 hm^2^	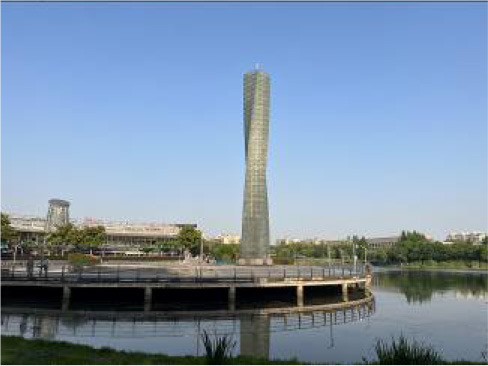	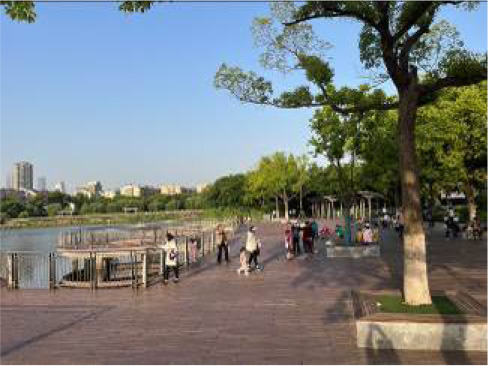
Bailuzhou Park	15 hm^2^	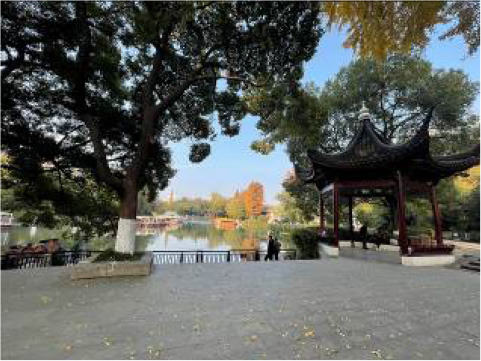	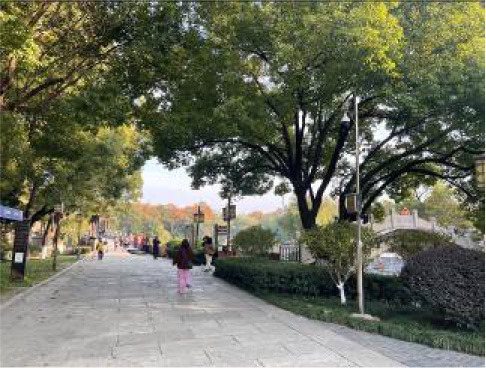
Zhenghe Park	2 hm^2^	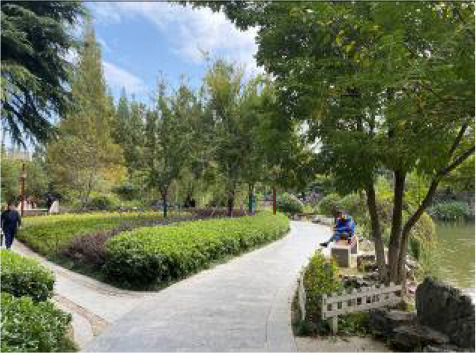	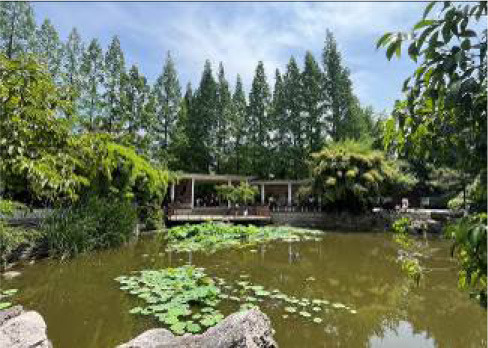

### 2.4 Data collection

From September 5 to September 30, 2024, the research team conducted face-to-face interviews with individuals aged 60 and older in five UGSs in sequence. Surveys were administered on weekdays and weekends, each session lasting approximately 3 h. The survey was conducted in real park environments, which allowed respondents to have a multi-sensory immersive experience and enabled the survey results to reflect the actual situation of their mental restoration in UGSs (22).

Initially, surveyors observed older adults in the UGSs before approaching them to explain the research and invite them to participate. Participants who consented completed the questionnaire within 8–12 min. A total of 420 individuals were surveyed, resulting in 389 valid questionnaires and a 93% validity rate. The questionnaires and interviews were administered in Chinese, with subsequent translation into English for publication. Participants were thoroughly informed about the research and signed an informed consent form prior to participation. Ethics approval was obtained from the Scientific Research Special Committee of Nanjing Tech University (approval number: NTJTECH-1-11).

The questionnaire comprised three sections. The first collected demographic information, including gender, age, education level, frequency of park visits, and typical visit duration. The second section used the eight factors of the PSDs to create a scale for evaluating the perceived qualities of UGSs. The third employed the simplified PRS to assess mental restoration among older adults. To accurately assess the effect of mental restoration in green spaces, respondents were prompted to recall their most distressing recent experience to elevate stress levels before completing the third part of the PRS questionnaire. This method has demonstrated efficacy in previous studies (58, 59).

### 2.5 Data analysis

All statistical analyses were conducted using IBM SPSS Statistics 26. We first assessed the reliability and validity of the 389 qualified questionnaires. According to Landis and Koch (1977), a Cronbach's α > 0.7 indicates a high level of reliability for a dataset (60). In this study, the PSDs scale (α = 0.777) and the PRS (α = 0.714) demonstrated satisfactory internal consistency. The questionnaire's structural validity was subsequently examined using KMO (Kaiser–Meyer–Olkin) and Bartlett's test of sphericity. KMO compares the magnitudes of observed correlation coefficients with those of partial correlation coefficients. The Bartlett test assesses the hypothesis that the correlation matrix of variables is an identity matrix, in which each variable correlates only with itself. A significant result indicates rejection of the null hypothesis. According to Tabachnick and Fidell (2012), KMO > 0.60 and a significant Bartlett test of sphericity (*p* < 0.05) indicate satisfactory factor analysis (61). The results demonstrate that the PSDs scale (KMO = 0.838, *p* < 0.001) and the PRS (KMO = 0.747, *p* < 0.001) exhibit satisfactory structural validity, rendering them suitable for further analysis.

A series of multivariate linear regression analyses was conducted to investigate the effects of UGS PSDs on the mental restoration of older adults, while controlling for covariates such as gender, age, education level, frequency of green space use, and duration of stay. To further explore gender-specific differences, stratified analyses were performed by separating participants based on gender, assessing the distinct impacts of the PSDs on mental restoration in older men and women. Additionally, respondents were categorized into three age groups−60–69 years, 70–79 years, and 80 years and above—to explore age-specific differences in how PSDs influence mental restoration. Regarding collinearity, all variance inflation factors ranged from 1.036 to 1.806, indicating no significant multicollinearity among the variables.

## 3 Results

### 3.1 Descriptive characteristics of respondents

As illustrated in Table 4, the proportions of male and female interviewees were comparable. Regarding age distribution, there was a slight predominance of individuals aged 70–79 years and a relative scarcity of those aged 80 years and older. In terms of education level, the largest proportion of respondents had completed senior high school (including technical secondary school), while the smallest proportion had attended elementary school or less. Regarding the frequency of green space use, the largest proportion reported visiting these spaces 5 or more times per week, whereas the smallest proportion reported visiting them less than once per week. For the duration of their stay, most respondents reported spending more than 2 h per visit in a green space, while the smallest proportion reported stays of less than 30 min.

**Table 4:** Characteristics of the interviewees.

**Characteristic**	**Category**	***N* = 389**
Gender	Men	49%
	Women	51%
Age	60–69	37%
	70–79	42%
	80 and above	21%
Education level	Elementary school and below	19%
	Junior high school	24%
	Senior high school (including technical secondary school)	32%
	College and above	25%
Frequency of green space use	Less than 1 time per week	7%
	1–2 times per week	13%
	3–4 times per week	14%
	5 or more times per week	66%
Duration of stay	30 min or less	6%
	30 min to 1 h	18%
	1–2 h	33%
	More than 2 h	43%

### 3.2 Effects of PSDs on the mental restoration of older adults

As illustrated in Table 5, culture, social, serene, and rich in species had a significant positive effect on mental restoration among older adults. Specifically, culture was positively correlated with fascination (*B* = 0.104, SE = 0.049, β = 0.162, 95% CI = 0.039–0.169), being-away (*B* = 0.133, SE = 0.035, β = 0.202, 95% CI = 0.064–0.201), and scope (*B* = 0.101, SE = 0.039, β = 0.142, 95% CI = 0.025–0.177). Social was positively correlated with fascination (*B* = 0.117, SE = 0.035, β = 0.173, 95% CI = 0.049–0.185), being away (*B* = 0.087, SE = 0.037, β = 0.125, 95% CI = 0.015–0.159), and scope (*B* = 0.088, SE = 0.041, β = 0.116, 95% CI = 0.008–0.168). Richness in species was positively correlated with fascination (*B* = 0.108, SE = 0.035, β = 0.160, 95% CI = 0.040–0.177) and being away (*B* = 0.078, SE = 0.037, β = 0.113, 95% CI = 0.006–0.151). Serene was positively correlated with fascination (*B* = 0.073, SE = 0.029, β = 0.134, 95% CI = 0.015–0.131) and being-away (*B* = 0.110, SE = 0.031, β = 0.196, 95% CI = 0.049–0.171).

**Table 5:** Regression analysis results of PSDs on mental restoration of older adults.

**Variable**	**Fascination**		**Being-away**		**Coherence**		**Scope**	
	***B*** **(SE)**	β	***B*** **(SE)**	β	***B*** **(SE)**	β	***B*** **(SE)**	β
(Constant)	1.394 (0.323)		2.089 (0.342)		2.763 (0.364)		2.103 (0.380)	
Nature	0.030 (0.047)	0.034	−0.048 (0.05)	−0.053	−0.049 (0.053)	−0.055	−0.017 (0.055)	−0.017
Culture	0.104^**^ (0.033)	**0.162** ^**^	0.133^***^ (0.035)	**0.202** ^***^	0.051 (0.037)	0.079	0.101^*^ (0.039)	**0.142** ^*^
Prospect	0.039 (0.036)	0.058	−0.022 (0.038)	−0.032	0.043 (0.040)	0.064	0.034 (0.042)	0.046
Social	0.117^**^ (0.035)	**0.173** ^**^	0.087^*^ (0.037)	**0.125** ^*^	0.025 (0.039)	0.037	0.088^*^ (0.041)	**0.116** ^*^
Space	−0.025 (0.033)	−0.041	−0.013 (0.035)	−0.020	0.007 (0.037)	0.011	0.056 (0.039)	0.082
Rich in species	0.108^**^ (0.035)	**0.160** ^**^	0.078^*^ (0.037)	**0.113** ^*^	0.061 (0.039)	0.090	0.048 (0.041)	0.064
Refuge	0.060 (0.040)	0.075	0.060 (0.042)	0.073	0.087 (0.045)	0.108	0.056 (0.047)	0.064
Serene	0.073^*^ (0.029)	**0.134** ^*^	0.110^***^ (0.031)	**0.196** ^***^	0.020 (0.033)	0.036	0.051 (0.035)	0.085
Gender	0.108 (0.065)	0.077	−0.043 (0.069)	−0.030	0.063 (0.073)	0.044	0.117 (0.077)	0.074
Age	−0.030 (0.044)	−0.031	0.024 (0.046)	0.025	0.114^*^ (0.049)	**0.118** ^*^	−0.025 (0.052)	−0.023
Education level	0.044 (0.027)	0.076	−0.010 (0.028)	−0.017	−0.008 (0.030)	−0.014	−0.084^**^ (0.031)	**−0.130** ^**^
Frequency of green space use	0.040 (0.035)	0.055	0.021 (0.037)	0.027	0.029 (0.039)	0.039	0.036 (0.041)	0.044
Duration of stay	0.029 (0.037)	0.037	0.112^**^ (0.039)	**0.140** ^**^	0.008 (0.041)	0.010	0.063 (0.043)	0.073
F	**9.906** ^***^		**7.808** ^***^		**2.462** ^**^		**6.130** ^***^	
Adjusted R^2^	0.230		0.186		0.047		0.147	

(1) B: Unstandardized coefficients, SE: Standard error, β: Standardized coefficients. (2) Bold indicates a significant relationship. (3) ^*^p < 0.05, ^**^p < 0.01, ^***^p < 0.001.

Both cultural and social factors had a positive impact on the three dimensions of mental restoration, while rich in species and serene had a positive impact on two of these dimensions. This indicated that UGSs' cultural and social characteristics generally had a greater impact on mental restoration among older adults. In terms of the β values, culture and serene had the strongest effect on being away, which indicated UGSs with cultural elements and a tranquil environment enabled older adults to temporarily forget the troubles of reality. Meanwhile, social and species-rich UGSs had the strongest effect on fascination, suggesting that UGSs with social activities and diverse species attracted the attention of older adults more effectively. In addition, age was positively correlated with coherence (*B* = 0.114, SE = 0.049, β = 0.118, 95% CI = 0.016–0.211). Education level was negatively correlated with scope (*B* = −0.084, SE = 0.031, β = −0.130, 95% CI = −0.146 to −0.023), and duration of stay was positively correlated with being away (*B* = 0.112, SE = 0.039, β = 0.140, 95% CI = 0.036–0.188).

### 3.3 Effects of PSDs on mental restoration of older adults of different genders

As illustrated in Table 6, stratified analyses by gender revealed that culture, rich in species, and serene had a positive effect on mental restoration across genders. For men, culture was positively correlated with being away (*B* = 0.163, SE = 0.053, β = 0.236, 95% CI = 0.058–0.268) and scope (*B* = 0.109, SE = 0.053, β = 0.162, 95% CI = 0.004–0.214). Rich in species was positively correlated with fascination (*B* = 0.105, SE = 0.051, β = 0.162, 95% CI = 0.004–0.205), being away (*B* = 0.121, SE = 0.056, β = 0.173, 95% CI = 0.011–0.231), and scope (*B* = 0.113, SE = 0.056, β = 0.165, 95% CI = 0.003–0.223). Serene was positively correlated with being away (*B* = 0.126, SE = 0.048, β = 0.223, 95% CI = 0.032–0.220). For women, culture was positively correlated with fascination (*B* = 0.152, SE = 0.047, β = 0.238, 95% CI = 0.058–0.246) and being away (*B* = 0.109, SE = 0.049, β = 0.174, 95% CI = 0.011–0.206). Rich in species was positively correlated with fascination (*B* = 0.104, SE = 0.050, β = 0.144, 95% CI = 0.006–0.202). Serene was positively correlated with fascination (*B* = 0.106, SE = 0.042, β = 0.186, 95% CI = 0.023–0.189).

**Table 6:** Regression analysis results of PSDs on mental restoration of older adults by gender.

**Variable**	**Fascination**		**Being-away**		**Coherence**		**Scope**	
	***B*** **(SE)**	β	***B*** **(SE)**	β	***B*** **(SE)**	β	***B*** **(SE)**	β
**Men**								
(Constant)	1.955 (0.505)		2.155 (0.550)		3.351 (0.564)		2.760 (0.551)	
Nature	0.028 (0.074)	0.032	−0.136 (0.080)	−0.144	−0.062 (0.082)	−0.071	−0.130 (0.080)	−0.140
Culture	0.069 (0.049)	0.107	0.163^**^ (0.053)	**0.236** ^**^	0.059 (0.055)	0.092	0.109^*^ (0.053)	**0.162** ^*^
Prospect	0.065 (0.052)	0.098	−0.019 (0.057)	−0.027	0.059 (0.058)	0.089	0.062 (0.057)	0.088
Social	0.179^**^ (0.051)	**0.269** ^**^	0.096 (0.055)	0.134	0.038 (0.057)	0.057	0.094 (0.055)	0.134
Space	−0.060 (0.047)	−0.103	−0.028 (0.051)	−0.044	0.036 (0.052)	0.061	0.048 (0.051)	0.078
Rich in species	0.105^*^ (0.051)	**0.162** ^*^	0.121^*^ (0.056)	**0.173** ^*^	0.128 (0.057)	0.197	0.113^*^ (0.056)	**0.165** ^*^
Refuge	0.081 (0.059)	0.102	0.077 (0.064)	0.090	−0.000 (0.065)	−0.000	0.089 (0.064)	0.106
Serene	0.045 (0.044)	0.086	0.126^**^ (0.048)	**0.223** ^**^	−0.028 (0.049)	−0.053	0.009 (0.048)	0.017
Age	−0.049 (0.070)	−0.047	−0.009 (0.076)	−0.008	0.097 (0.078)	0.093	−0.075 (0.076)	−0.068
Education level	−0.007 (0.043)	−0.011	−0.032 (0.046)	−0.046	−0.056 (0.047)	−0.086	−0.120^*^ (0.046)	**−0.174** ^*^
Frequency of green space use	−0.001 (0.055)	−0.002	0.035 (0.060)	0.041	−0.029 (0.061)	−0.036	0.004 (0.060)	0.005
Duration of stay	−0.016 (0.058)	−0.018	0.099 (0.063)	0.107	0.005 (0.065)	0.006	0.041 (0.064)	0.045
*F*	**5.686** ^***^		**5.150** ^***^		1.568		**4.270** ^***^	
Adjusted *R*^2^	0.229		0.209		0.035		0.172	
**Women**								
(Constant)	1.176 (0.439)		2.083 (0.456)		2.521 (0.497)		1.756 (0.553)	
Nature	0.035 (0.063)	0.039	0.028 (0.066)	0.031	−0.028 (0.072)	−0.030	0.080 (0.080)	0.075
Culture	0.152^**^ (0.047)	**0.238** ^**^	0.109^*^ (0.049)	**0.174** ^*^	0.028 (0.054)	0.042	0.107 (0.060)	0.142
Prospect	0.020 (0.051)	0.030	−0.025 (0.053)	−0.038	0.046 (0.058)	0.067	0.026 (0.064)	0.033
Social	0.063 (0.049)	0.091	0.068 (0.05)	0.100	0.011 (0.055)	0.016	0.073 (0.061)	0.089
Space	0.005 (0.049)	0.008	0.023 (0.051)	0.036	−0.025 (0.056)	−0.038	0.076 (0.062)	0.100
Rich in species	0.104^*^ (0.050)	**0.144** ^*^	0.017 (0.052)	0.024	−0.001 (0.057)	−0.001	−0.022 (0.063)	−0.026
Refuge	0.014 (0.058)	0.017	0.064 (0.060)	0.083	0.160^*^ (0.066)	**0.197** ^*^	0.013 (0.073)	0.014
Serene	0.106^*^ (0.042)	**0.186** ^*^	0.082 (0.044)	0.148	0.047 (0.048)	0.081	0.092 (0.053)	0.137
Age	−0.022 (0.057)	−0.025	0.043 (0.059)	0.050	0.119 (0.065)	0.133	0.011 (0.072)	0.010
Education level	0.085 (0.034)	0.157	0.004 (0.036)	0.007	0.026 (0.039)	0.047	−0.061 (0.043)	−0.096
Frequency of green space use	0.056 (0.047)	0.082	0.009 (0.049)	0.013	0.071 (0.053)	0.102	0.053 (0.059)	0.066
Duration of stay	0.057 (0.048)	0.081	0.121^*^ (0.050)	**0.176** ^*^	0.000 (0.054)	0.000	0.069 (0.06)	0.083
*F*	**5.953** ^***^		**3.400** ^***^		**2.087** ^*^		**3.293** ^***^	
Adjusted *R*^2^	0.231		0.127		0.062		0.122	

(1) B: Unstandardized coefficients, SE: Standard error, β: Standardized coefficients. (2) Bold indicates a significant relationship. (3) ^*^p < 0.05, ^**^p < 0.01, ^***^p < 0.001.

According to the research findings, culture influenced men and women in two dimensions, respectively, while serene influenced both genders in one dimension. This indicates that the impact of these two PSDs on the mental restoration of different genders was not significantly different. Rich in species affected men in three dimensions but only influenced women in one dimension. This indicates that although UGSs with rich species had a restorative effect on both men and women, they could produce a more diverse range of restorative effects for men.

Nevertheless, there were notable differences as well. Social had a positive effect on men, while refuge had a positive influence on women. For men, social was positively correlated with fascination (*B* = 0.179, SE = 0.051, β = 0.269, 95% CI = 0.079–0.279). For women, refuge was positively correlated with coherence (*B* = 0.160, SE = 0.066, β = 0.197, 95% CI = 0.031–0.290). In addition, for men, education level was negatively correlated with scope (*B* = −0.120, SE = 0.046, β = −0.174, 95% CI = −0.211–0.028). For women, duration of stay was positively correlated with being away (*B* = 0.121, SE = 0.050, β = 0.176, 95% CI = 0.023–0.219).

### 3.4 Effects of PSDs on the mental restoration of older adults of different ages

As illustrated in Table 7, stratified analysis by age indicated that culture, social, and serene were positively correlated with mental restoration among those aged 60–69 and 70–79 years. For individuals aged 60–69 years, culture was positively correlated with fascination (*B* = 0.143, SE = 0.055, β = 0.230, 95% CI = 0.039–0.169) and being-away (*B* = 0.134, SE = 0.053, β = 0.219, 95% CI = 0.029–0.239). Social was positively correlated with being away (*B* = 0.132, SE = 0.057, β = 0.191, 95% CI = 0.019–0.244). Serene was positively correlated with being away (*B* = 0.137, SE = 0.050, β = 0.239, 95% CI = 0.038–0.237). For individuals aged 70–79 years, culture was positively correlated with being-away (*B* = 0.133, SE = 0.055, β = 0.194, 95% CI = 0.024–0.242) and scope (*B* = 0.172, SE = 0.060, β = 0.230, 95% CI = 0.054–0.291). Social was positively correlated with fascination (*B* = 0.166, SE = 0.048, β = 0.276, 95% CI = 0.071–0.262). Serene was positively correlated with being away (*B* = 0.112, SE = 0.050, β = 0.207, 95% CI = 0.013–0.210). Additionally, education level and frequency of green space use had a significant effect on mental restoration among those aged 60–69 and 70–79 years. For individuals aged 60–69 years, education level was positively correlated with fascination (*B* = 0.115, SE = 0.052, β = 0.169, 95% CI = 0.012–0.219). Frequency of green space use was positively correlated with being away (*B* = 0.118, SE = 0.053, β = 0.172, 95% CI = 0.013–0.222). For individuals aged 70–79 years, education level was negatively correlated with scope (*B* = −0.152, SE = 0.049, β = −0.224, 95% CI = −0.249 to −0.054). Frequency of green space use was negatively correlated with being away (*B* = −0.129, SE = 0.059, β = −0.160, 95% CI = −0.246 to −0.012).

**Table 7:** Regression analysis results of PSDs of UGSs on mental restoration among older adults by age group.

**Variable**	**Fascination**		**Being-away**		**Coherence**		**Scope**	
	***B*** **(SE)**	β	***B*** **(SE)**	β	***B*** **(SE)**	β	***B*** **(SE)**	β
**60–69 years**								
(Constant)	1.025 (0.547)		2.087 (0.523)		2.533 (0.594)		1.876 (0.601)	
Nature	−0.048 (0.089)	−0.051	−0.161 (0.085)	−0.176	−0.122 (0.096)	−0.134	0.028 (0.097)	0.030
Culture	0.143^*^ (0.055)	**0.230** ^*^	0.134^*^ (0.053)	**0.219** ^*^	0.041 (0.06)	0.068	0.036 (0.061)	0.056
Prospect	0.090 (0.063)	0.125	−0.082 (0.06)	−0.116	0.035 (0.068)	0.050	0.027 (0.069)	0.036
Social	0.061 (0.06)	0.087	0.132^*^ (0.057)	**0.191** ^*^	0.011 (0.065)	0.016	0.059 (0.065)	0.082
Space	−0.060 (0.069)	−0.080	0.060 (0.066)	0.081	−0.088 (0.075)	−0.120	0.136 (0.076)	0.178
Rich in species	0.180^**^ (0.063)	**0.246** ^**^	0.108 (0.06)	0.150	0.133 (0.068)	0.185	0.080 (0.069)	0.106
Refuge	0.059 (0.073)	0.071	0.064 (0.070)	0.078	0.153 (0.080)	0.188	0.073 (0.081)	0.086
Serene	0.062 (0.053)	0.106	0.137^**^ (0.05)	**0.239** ^**^	0.062 (0.057)	0.108	0.009 (0.058)	0.014
Gender	0.085 (0.121)	0.056	0.021 (0.115)	0.014	0.040 (0.131)	0.027	0.189 (0.133)	0.121
Education level	0.115^*^ (0.052)	**0.169** ^*^	−0.084 (0.050)	−0.125	0.016 (0.057)	0.024	−0.101 (0.057)	−0.145
Frequency of green space use	0.058 (0.055)	0.083	0.118^*^ (0.053)	**0.172** ^*^	0.021 (0.060)	0.030	0.068 (0.061)	0.095
Duration of stay	0.056 (0.063)	0.072	0.102 (0.061)	0.133	0.135 (0.069)	0.178	0.044 (0.070)	0.055
*F*	**4.803** ^***^		**5.619** ^***^		**1.868** ^*^		**2.721** ^**^	
Adjusted *R*^2^	0.234		0.281		0.068		0.127	
**70–79 years**								
(Constant)	2.122 (0.484)		2.920 (0.527)		3.486 (0.550)		2.255 (0.573)	
Nature	0.127 (0.075)	0.147	−0.063 (0.081)	−0.067	0.061 (0.085)	0.069	−0.007 (0.088)	−0.006
Culture	0.014 (0.051)	0.022	0.133^*^ (0.055)	**0.194** ^*^	−0.019 (0.058)	−0.029	0.172^**^ (0.060)	**0.230** ^**^
Prospect	0.008 (0.055)	0.013	0.044 (0.060)	0.068	0.019 (0.063)	0.031	0.089 (0.065)	0.124
Social	0.166^**^ (0.048)	**0.276** ^**^	0.041 (0.053)	0.061	0.039 (0.055)	0.064	0.086 (0.057)	0.118
Space	−0.026 (0.046)	−0.050	−0.032 (0.051)	−0.057	0.051 (0.053)	0.097	0.022 (0.055)	0.035
Rich in species	0.047 (0.054)	0.073	0.115 (0.059)	0.163	0.059 (0.061)	0.090	0.086 (0.064)	0.112
Refuge	0.021 (0.057)	0.030	0.016 (0.063)	0.020	0.041 (0.065)	0.057	−0.012 (0.068)	−0.014
Serene	0.088 (0.046)	0.179	0.112^*^ (0.050)	**0.207** ^*^	−0.066 (0.052)	−0.133	0.026 (0.054)	0.044
Gender	0.006 (0.098)	0.005	−0.096 (0.107)	−0.067	0.080 (0.111)	0.060	0.150 (0.116)	0.096
Education level	−0.006 (0.042)	−0.011	−0.008 (0.045)	−0.013	−0.028 (0.047)	−0.049	–0.152^**^ (0.049)	–0.224^**^
Frequency of green space use	−0.025 (0.054)	−0.033	–0.129^*^ (0.059)	–0.160^*^	0.026 (0.062)	0.035	−0.035 (0.064)	−0.040
Duration of stay	0.044 (0.057)	0.060	0.086 (0.062)	0.106	−0.045 (0.064)	−0.060	0.090 (0.067)	0.101
*F*	**3.827** ^***^		**4.013** ^***^		0.551		**4.292** ^***^	
Adjusted *R*^2^	0.171		0.181		−0.034		0.194	

(1) B: Unstandardized coefficients, SE: Standard error, β: Standardized coefficients. (2) Bold indicates a significant relationship. (3) ^*^p < 0.05, ^**^p < 0.01, ^***^p < 0.001. (4) Findings for the group aged 80 and above are excluded due to the absence of significant outcomes.

The effects of culture, social, and serene on mental restoration showed no significant differences across different age groups. Rich in species exerted a positive effect only on individuals aged 60–69 years and was positively correlated with fascination (*B* = 0.180, SE = 0.063, β = 0.246, 95% CI = 0.056–0.304). No significant effect of PSDs on individuals aged 80 years and above was observed.

## 4 Discussion

According to the study findings, the following PSDs positively influence mental restoration in older adults: culture, social, rich in species, and serene. From the perspective of gender differences, social only exerts a positive effect on men, while refuge only has a positive impact on women. From an age-group perspective, cultural, social, and serene environments contribute positively to mental restoration among individuals aged 60–79 years, whereas species richness exerts a positive effect only among those aged 60–69 years (Table 8). It should be noted that this study employed a cross-sectional design, so the observed associations cannot yet serve as evidence of a causal relationship. Nevertheless, the results still demonstrate the impact of environmental attributes on the mental health of older adults, as well as age- and gender-based differences in these impacts. The findings contribute to the development of more equitable and inclusive environmental strategies for the “promotion of mental health and wellbeing” proposed in SDG 3.

**Table 8:** Impact of PSDs on mental restoration in different studied populations.

**Variable**	**Older adults**	**Men**	**Women**	**60–69 year old**	**70–79 year old**
Culture	√	√	√	√	√
Social	√	√		√	√
Rich in species	√	√	√	√	
Serene	√	√	√	√	√
Refuge			√		

√ indicates that the PSD has a positive effect on mental restoration.

### 4.1 PSDs that promote mental restoration in older adults

Addressing study objective 1, PSDs (cultures, social, rich in species, and serene) positively influenced mental restoration in older adults. This result bears similarities to the research findings in other countries. In their research in southern Sweden, Jong et al. identified culture, serene, and richness in species as three characteristics beneficial to mental health (62). A study in Japan, on the other hand, found that serene and richness in species are two key characteristics conducive to mental restoration (50). In an earlier study conducted in Copenhagen, social and serene were found to be significantly correlated with mental restoration among general green space users (20).

Nevertheless, culture and society were seldom identified as having a positive effect on mental restoration in previous studies. Indeed, some research indicated that social interaction negatively impacted recovery in certain contexts (63). Notably, earlier studies did not focus on older adults, necessitating a discussion of the present findings within the framework of older adult characteristics.

The culture dimension of UGSs is primarily reflected in the decorative elements and ornamental features found within green spaces, such as statues, fountains, pavilions, and exotic plants. These elements have a restorative effect, observable in two aspects. First, the cultural connotations embedded within these landscape features enrich the spiritual lives of older adults, who often experience a sense of spiritual emptiness as they withdraw from social production (64). The integration of cultural elements can enhance the humanistic quality of these spaces, stimulate the senses, and fulfill spiritual needs (65). Second, the placement of these features often provides places for older adults to rest, relax, and engage in social interaction. They can cultivate neighborhood attachment, interaction, and community participation through outdoor spaces, thereby increasing mutual contact, understanding, and psychological comfort among older adults (25).

The social dimension of UGSs plays a crucial role in the mental restoration of older adults. Chen et al. found that social was the most easily perceived environmental characteristic in UGSs in China, whereas it was the least perceived in European UGSs (45). This phenomenon may be attributed to the extremely high urban population density in China. UGSs with open areas and fresh air have become primary venues for social activities among Chinese residents, especially older adults. In contrast, in European countries with smaller populations, it may be difficult to access the large number of social activities common in Chinese UGSs.

According to the socioemotional selectivity theory, older adults become increasingly aware of their limited time (66). They seek to fulfill their higher emotional needs and attain greater life satisfaction through social interactions with emotionally close partners, alleviating the physical and mental stress associated with aging (67). Participation in social activities allows older adults to recognize their value through meaningful roles, fostering a sense of achievement and participation. This engagement, in turn, helps alleviate the anxiety and stress often associated with retirement and the challenges of aging, promoting both emotional wellbeing and mental restoration (68). Empirical studies indicate that socially oriented activities, such as playing chess and cards or engaging in conversation, rank among the most common outdoor activities among older adults in China (69–71). It can be concluded that social interaction positively affects the mental health of older adults. UGSs provide not only venues for physical activity but also settings for social interaction (72). This evidence supports the assertion that the social dimension of UGSs positively influences mental restoration.

Numerous studies have previously identified the natural dimension of UGSs as significant for facilitating mental restoration (73, 74). However, this was not evident in the present study. This discrepancy may arise from how the natural dimension is defined within the PSDs, which focus on environments with a wild, untouched quality. Given the physical and mental deterioration often experienced by older adults, unmanaged natural environments may evoke feelings of insecurity, thereby detracting from their attractiveness and potentially hindering mental restoration (75).

Despite these findings, it does not imply that nature has no effect on mental restoration in older adults. The species richness dimension, which denotes biodiversity within UGSs, represents the natural dimension. The present study found that environments rich in species have a positive effect on both fascination and a sense of being away. On the one hand, a rich diversity of species enhances environmental appeal, possibly reducing directed-attention fatigue. The variety and complexity inherent in biodiverse spaces likely stimulate interest and curiosity, supporting mental engagement and restoration from cognitive overload (76). On the other hand, the abundant plants in UGSs are typically maintained artificially, providing older adults with a greater sense of security than unmanaged natural environments, allowing them to relax both physically and psychologically while experiencing enhanced restoration (77).

The contribution of the serene dimension to mental restoration has been consistently confirmed, indicating that serene environments are generally restorative for individuals across various demographics (78–80). However, a minority of studies have found a negative correlation between serene and recovery, primarily due to insecurity stemming from inadequate environmental maintenance (81). Hence, the level of maintenance is closely tied to the restorative effects of UGSs. Only when a serene environment is combined with optimal maintenance can it effectively promote the mental restoration in older adults (82).

### 4.2 Differences in the restorative effects of UGSs on older adults by gender

Regarding study objective 2, the findings highlight differences in the restorative effects of UGSs on older adults across genders: social interactions demonstrated a positive effect only for men, and refuge exclusively benefited women. Research indicates that men generally exhibit a greater inclination toward socialization, with a higher proportion of older men engaging in social activities compared to their female counterparts. Activities involving interaction have been associated with higher levels of wellbeing among older men (83). Therefore, it may be argued that the social dimension of UGSs provides a more restorative experience for men, as social engagement may more effectively fulfill their emotional and psychological needs. Conversely, older women tend to report greater wellbeing when participating in solitary activities at home (84). Consequently, UGSs that incorporate social features may be more beneficial for the mental restoration of older men.

The refuge dimension uniquely contributes to mental restoration for older women, possibly due to the heightened prevalence of mental health issues among this demographic. Surveys across numerous countries indicate a higher prevalence of depressive symptoms in women compared to men (85). In China, for example, the number of women with depression is approximately 50% higher than that of men (86). Furthermore, the cumulative disadvantage theory posits that early-life disadvantages experienced by women—such as lower education levels, decreased income, limited social capital, and discriminatory experiences—accumulate over time. This accumulation of disadvantages contributes to a widening mental health gap between genders as they age (87). Several studies have indicated that Chinese women experience significant disparities in mental health outcomes compared to men, with these discrepancies increasing with age (88). Consequently, older women in China face a higher risk of developing mental health issues compared to men (89). In addition, most older women in China bear the responsibility of taking care of their families and spouses, which may increase their psychological distress (90).

The refuge dimension encompasses opportunities for individuals to occupy small, secure areas surrounded by vegetation (38). This dimension not only provides rest but also aids in emotional regulation (91). To mitigate psychological distress, older women often seek environments that convey a sense of security and refuge. The pronounced influence of refuge quality on the coherence dimension suggests that spaces that offer enclosure and perceived safety better align with the restorative preferences of older women.

### 4.3 Differences in the restorative effects of UGSs on older adults by age group

Regarding study objective 3, the findings demonstrate that while cultural, social, and serene environmental dimensions positively influence mental restoration in individuals aged 60–79 years, environments rich in species positively affect mental restoration only in those aged 60–69 years. This difference may be due to age-related declines in sensory and cognitive abilities.

At least three theoretical models have been proposed to explain age-related changes in sensory and cognitive processing: the common cause hypothesis, the sensory deprivation hypothesis, and the information degradation hypothesis. The common cause hypothesis posits that a domain-general mechanism accounts for a substantial portion of the age-related decline in sensory and cognitive functions, thereby yielding high concurrent validity (92). The sensory deprivation hypothesis posits that prolonged decline in perception gradually degrades neural substrates, affecting cognition (93). The information degradation hypothesis proposes that, because perceptual and cognitive processes rely on a shared resource-processing system, age-related perceptual decline results in a reallocation of limited cognitive resources to perceptual tasks, reducing the availability of cognitive resources for subsequent cognitive tasks and contributing to cognitive decline (94). Collectively, these theories indicate a demonstrable decline in sensory and cognitive functions as individuals age. Moreover, research indicates that after age 70, older adults enter a sensitive period of cognitive aging (95), during which memory decline accelerates (96).

Individuals aged 70–79 years typically exhibit a more pronounced decline in sensory and cognitive functions compared to those aged 60–69 years. Consequently, their ability to process information from their environment diminishes, rendering even abundant species less likely to capture their perceptual attention. This is likely the primary reason why richness in species exerts a restorative effect on individuals aged 60–69 years.

### 4.4 Advantages and limitations

To our knowledge, this study is among the first to examine the associations between the PSDs of UGSs and mental restoration in older adults. The findings enable decision-makers and practitioners to gain a clearer understanding of the relationship between green space characteristics and mental restoration in older adults, thereby helping them achieve more restorative experiences in UGSs and enjoy the “Good Health and WellBeing” required by SDG 3. By focusing on the relationship between UGSs and the mental health of older adults, this study also provides insights with both theoretical depth and practical value in the field of gerontology. These findings emphasize that, in gerontological research and urban design practice, attention should be paid to the diversity within older adults to develop strategies that are more equitable and inclusive.

However, this study has limitations that should be addressed in future research. First, it is a cross-sectional study, and the restorative characteristics of environments may change over time. For example, psychological responses elicited by park landscapes may vary by season. Future research should use longitudinal data and examine the impact of relationships over time to compare findings across seasons and observe evolution. Given that this study employs a clear and well-defined research design, as well as unified questionnaires and data formats, the replication of this research is feasible.

Second, all participants were drawn from UGSs, indicating prior interaction experience with such environments. While this provides respondents with a basis for forming questionnaire scores from direct experience, it limits the generalizability of the research findings due to the concentrated sample of green space users. Individuals who frequently use green spaces are likely to be more sensitive to green space environments and derive significant benefits from them. These samples only represent the group that benefits the most. The average restorative effect derived from these samples may be overestimated, leading to overly optimistic conclusions. Therefore, it is recommended that future studies on this topic be conducted by randomly sampling both green space users and non-users to encompass a broader range of respondents.

Finally, this survey focused on a single city. Given that the characteristics of green space and cultural backgrounds vary across regions, older adults may perceive the utilization and environmental preferences of UGSs differently. Future studies may select multiple cities with differing climates and cultural backgrounds for comparative analysis, thereby further verifying the generalizability of this study's conclusions.

## 5 Conclusion

In the PSDs of UGSs, four components—culture, social, rich in species, and serene—were found to have a positive effect on the mental restoration of older adults. From a gender perspective, culture, serene, and rich in species positively influenced mental restoration among older adults, while social factors positively affected older men, and refuge benefited older women. From an age perspective, culture, social, and serene positively influenced mental restoration among individuals aged 60–79 years, whereas species richness only positively affected those aged 60–69 years.

The findings of this study can inform UGSs' design in four key ways: (1) thoughtfully incorporating human-made elements such as fountains, statues, and pavilions can enhance aesthetic appeal and foster social interaction, thereby promoting emotional wellbeing among older adults. (2) Balancing social and quiet areas is essential to accommodating the diverse preferences of older adults, alongside maintaining UGSs to promote a sense of security. (3) Enhancing plant biodiversity by introducing diverse ornamental species can enrich sensory experiences, benefiting mental restoration, particularly as older adults retain some degree of perceptual plasticity despite age-related declines. (4) In response to the elevated mental health risks faced by older women, prioritizing refuge-like features in UGSs is crucial, including the introduction of moderate enclosures, improved spatial recognition, and maintained spatial connectivity to foster a sense of safety and comfort.

By analyzing the impact of urban green spaces on mental restoration in older adults, this study not only provides an operable environmental support pathway for SDG 3 but also offers empirical evidence for enriching environmental intervention strategies in the field of gerontology, thereby contributing to the development of age-friendly urban green spaces with greater inclusiveness and health-promoting functions. Future studies should incorporate a broader survey population, diverse geographical areas, and seasonal variations to yield comprehensive data.

## Data Availability

The datasets presented in this study can be found in online repositories. The names of the repository/repositories and accession number(s) can be found in the article/supplementary material.
